# Pulsed Electromagnetic Field (PEMF) Stimulation Increases Muscle Activity During Exercise in Sedentary People

**DOI:** 10.3390/jfmk10020232

**Published:** 2025-06-19

**Authors:** Aurelio Trofè, Alessandro Piras, Luca Breviglieri, Alessandra Laffi, Andrea Meoni, Milena Raffi

**Affiliations:** 1 Department for Life Quality Studies, University of Bologna, Corso D’Augusto 237, 47921 Rimini, Italy; aurelio.trofe2@unibo.it (A.T.); alessandro.piras3@unibo.it (A.P.); lucabreviglieri@gmail.com (L.B.); 2 Department of Biomedical and Neuromotor Sciences, University of Bologna, 40126 Bologna, Italy; alessandra.laffi3@unibo.it (A.L.); andrea.meoni@unibo.it (A.M.)

**Keywords:** EMG, cycling, muscle response amplitude, PEMF

## Abstract

**Objectives:** A pulsed electromagnetic field (PEMF) induces electric currents in biological tissue, enhancing muscle energy expenditure during heavy constant-load exercises. In this paper, we investigate the PEMF effect on muscular activation in male sedentary people. **Methods**: The surface electromyographic (EMG) activity of the right leg’s vastus medialis (RVM) and biceps femoris (RBF) muscles was recorded and analyzed. The root mean square values were normalized to the peak amplitude observed during maximal voluntary contraction. Measurements were taken at baseline (stationary seated position), during warm-up (unloaded cycling), and throughout 15 min of constant-load exercise performed at moderate intensity. Subjects performed two experimental conditions, when PEMF was turned ON versus OFF. **Results**: No significant difference was found during the baseline. The analysis during warm-up showed significant differences between conditions (ON vs. OFF) for both muscles (RVM *p* = 0.019; RBF *p* < 0.001). The analysis during constant-load exercise showed significant differences between conditions (ON vs. OFF) for RVM only (*p* = 0.002). **Conclusions**: This study provides evidence that PEMF stimulation acutely enhances muscle activation, primarily in the vastus medialis, with a comparatively smaller effect on the biceps femoris during moderate-intensity cycling in sedentary young men. The observed increase in EMG activity suggests that PEMF may facilitate neuromuscular excitability and muscle recruitment, potentially through mechanisms related to calcium signaling and enhanced muscle perfusion.

## 1. Introduction

Pulsed electromagnetic fields (PEMFs) refer to a form of therapy that utilizes electromagnetic fields to promote various physiological effects within the human body. Therapy has shown positive effects in managing pain, promoting tissue repair, and improving overall well-being [1].

PEMF therapy operates on the principle that electromagnetic fields can interact with biological systems at a cellular level. This interaction can influence cellular behavior, modulate biochemical processes, and trigger a range of beneficial effects [2].

Few studies have suggested that PEMF therapy may help in reducing pain and inflammation, accelerating bone healing, enhancing nerve regeneration, and improving circulation [3,4,5]. PEMF therapy has promising applications for the treatment of acute and chronic tissue inflammation [6,7]. PEMF could be a positive influence in the treatment of delayed-onset muscle soreness (DOMS) after exercise [8]. Authors have investigated PEMF therapy on physiological changes related to DOMS, like pain, soreness, or muscle force generation, in order to assess recovery after isometric exercise session. The application of PEMF treatment on the biceps brachii for ten minutes after training reduced the severity of perceived symptoms of DOMS in the following days, enhancing the quality of recovery. PEMF stimulation also raised the median frequency of muscle activation and reduced electromechanical delay during isometric contraction in the day after the exercise [8]. Despite that, no effect was found on the peak of isometric force generation.

While the exact mechanisms through which PEMF therapy exerts its effects are still unclear, several hypotheses have been proposed. It is believed that PEMF therapy can stimulate the production of various molecules and signaling pathways involved in tissue repair, such as growth factors, nitric oxide, and cytokines [9,10,11].

PEMF could increase blood flow and increase nitric oxide synthesis activity [9,12] and consequently enhance NO-cyclic guanosine monophosphate (cGMP) cascades, leading to vasodilation. The effect of PEMF was likely mediated through calcium/calmodulin (CaM)-dependent nitric oxide cascades [13]. PEMF probably enhances the binding of Ca^2+^ and calmodulin, and then, Ca^2+^CaM binds to e-NOS to release NO. Additionally, PEMF may modulate cellular membrane potentials, ion transport, and calcium signaling, all of which play crucial roles in cellular function [1].

Despite the considerable research and several uses for medical purposes, few studies have evaluated the effects of PEMF stimulation during exercise. Kim et al. [12] investigated the influence of 3 months of PEMF treatment on plasma nitric oxide (NO) in 23 subjects with mild to moderate metabolic syndrome. The authors showed that 16 min of stimulation three times/day were able to increase blood flow and circulating plasma NO levels at rest and at the end of submaximal exercise performed at moderate intensity.

Trofè et al. [14] investigated the effect of acute PEMF stimulation on VO_2_ and muscle oxygenation in twenty semi-professional cyclists. The authors found that stimulation applied during a heavy constant-load exercise was able to increase the rate of muscle oxygen extraction and utilization. Stimulation augmented the quantity and the velocity of muscle O_2_ available, accelerating HHb kinetics without affecting pulmonary VO_2_ on-transition kinetics. Moreover, Trofè et al. [15] investigated the PEMF effect on the amplitude of muscle activity in semi-professional cyclists. The stimulation was able to increase the amplitude of the muscle activity, of both the vastus medialis and biceps femoris, during warm-up, when the subjects cycled without load, at a low intensity. This effect could be due to changes in membrane permeability [16] and Ca^2+^ channel conduction [17]. Thus, it could increase ion flux and cellular concentrations [18,19], improving contraction mechanisms during exercise.

It has been hypothesized that PEMF could affect energetic metabolism, especially the glycolytic metabolism of type-II muscular fibers [20]. In our previous study [14], PEMF stimulation increased the blood lactate level during exercise as a consequence of a potential influence on the glycolytic metabolism of type-II fibers. We can hypothesize that PEMF caused a change in membrane permeability [16] and calcium channel conduction [17], augmenting contraction mechanisms during physical activity. A recent study showed that PEMF could affect glucose utilization [21]. In rats with streptozotocin-induced diabetic muscle atrophy, PEMF stimulation influenced metabolic enzymes in the quadriceps, suggesting an increase in the metabolic capacity of muscle.

In insulinoma cells, PEMF stimulation attenuated insulin secretion, probably due to an influence on ions flux and Ca^2+^ channels [20]. Considering all these premises, the investigation of PEMF effects during exercise, and more precisely, assessing its influence on muscle activity is important. We hypothesize that PEMF stimulation could improve muscle response following its implication on the mechanisms involved in muscular contraction. Therefore, the purpose of this study was to evaluate the effect of PEMF on young sedentary people during a constant-load exercise performed at moderate intensity.

## 2. Materials and Methods

The study followed a single-blind, randomized controlled trial design (ClinicalTrials.gov ID: NCT06446466). Nine sedentary young men participated in the study (Table 1). Based on an effect size of 0.45 reported by Trofè et al. [14,15], a power analysis using the G*Power software version 3.1.9.2 (Kiel, Germany) indicated that a total sample size of eight would provide sufficient statistical power (0.80) to detect a significant difference at an alpha level of 0.05. One additional participant was included to account for potential missing or corrupted data.

### 2.1. Participants

Participant recruitment focused on healthy, sedentary young male volunteers, ensuring homogeneity in baseline physical condition. The inclusion criteria were male, sedentary habits, age <35 yo, non-smoker. The exclusion criteria were: neuro-muscular injuries, physical deficits, mental illness, medication, or supplement use. Each participant received both written and verbal explanations of the experimental procedures, and informed consent was obtained prior to data collection. The study was conducted in accordance with the Declaration of Helsinki and was approved by the Bioethics Committee of the University of Bologna (protocol code: 23212, approval date: 5 February 2020).

**Table 1:** Descriptive characteristics of the study participants. Data are presented for each subject, including age, maximal oxygen consumption (VO_2_ max, expressed in mL/min/kg), workload at exhaustion (watts), body weight (kg), height (cm), and body mass index (BMI). Mean values, standard deviations (SD), and standard errors of the mean (SEM) are also reported.

Subjects	Age (Years)	VO_2_ max (mL/min/kg)	Workload (Watt)	Weight (kg)	Height (cm)	BMI
L.B	32	33.9	108	76.0	175	24.8
E.M	30	31.9	113	72.0	180	22.2
A.T	34	33.2	100	77.0	177	24.6
S.B	34	33.3	108	87.0	178	27.5
M.S	23	34.4	88	62.0	170	21.5
A.A	20	33.9	85	67.5	173	22.6
S.P	25	39.5	105	64.0	164	23.8
E.B	35	30.5	83	79.0	173	26.4
S.I	32	35.3	106	74.0	168	26.2
Mean	29.4	34.0	99.6	73.2	173.1	24.4
SD	5.4	2.5	11.3	7.8	5.1	2.1
SEM	1.8	0.8	3.8	2.6	1.7	0.7

### 2.2. EMG and PEMF Recordings

The experiments were conducted using a cycle ergometer (H-300-R Lode, Exere Air Machine, Cesena, Italy), following a standardized protocol in a quiet room maintained at a temperature of 22 °C. To minimize circadian variability, all recordings were performed consistently between 9:00 a.m. and 12:00 p.m. Participants were instructed to abstain from caffeine, alcohol, and strenuous physical activity for 12 h prior to the experiment.

EMG recordings: We recorded surface EMG activity from the right vastus medialis (RVM) and right biceps femoris (RBF) caput longum at a sampling rate of 1000 Hz by a Free-EMG 1000 (BTS Bioengineering, Inc. Garbagnate Milanese, Italy). Electrodes were placed on the muscular belly of the RVM and RBF, according to SENIAM guidelines (www.seniam.org, accessed on 13 February 2023). To improve contact, the skin was shaved and cleaned with ethanol before placing the Ag/AgC1 disposable electrodes (32 × 32 mm with an active area of 0.8 cm^2^), and an inter-electrode distance of 2 cm was used in the bipolar configuration (RAM, s.r.l, Genova, Italy). Free-EMG is a device with wireless miniaturized probes for the dynamic analysis of muscle activity. The system communicates with a PC through the supplied USB 2.0 receivers with wireless IEEE802.15.4 data transmission. For signal acquisition, the probes (41.5 × 24.8 × 14 mm) are directly attached to the electrodes, and signals are detected and captured. Each probe is equipped with internal memory to ensure uninterrupted recording in the case of temporary connection loss.

**Figure 1:**
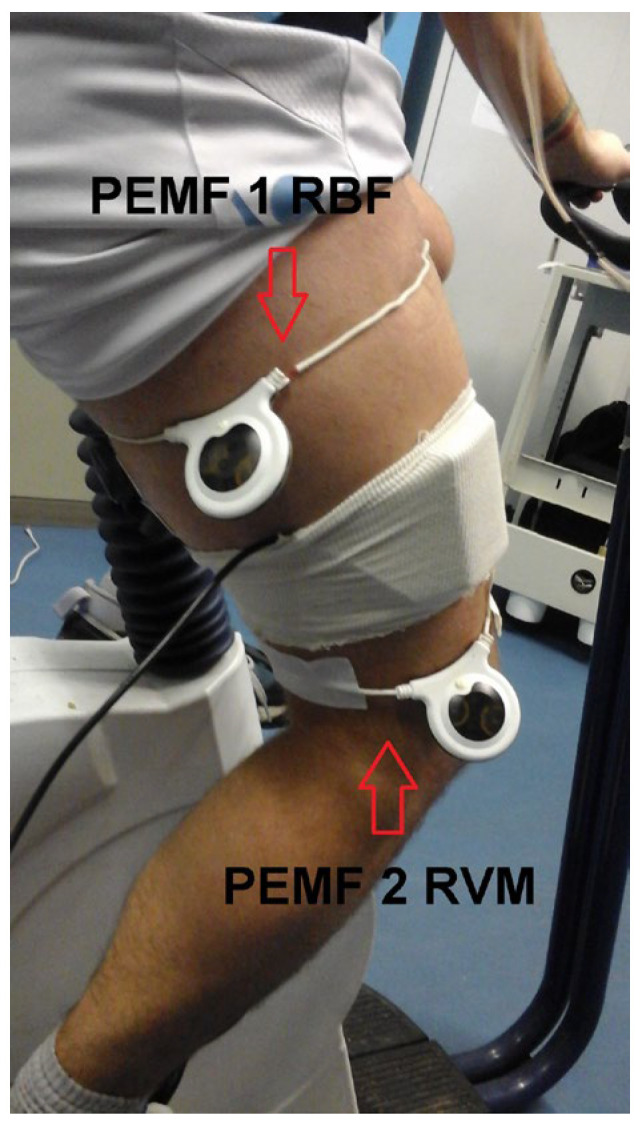
Experimental setup. The two circular 20 cm PEMF loop-antenna devices were positioned on the right thigh, at the level of the belly of the vastus medialis and biceps femoris caput longum. The figure also shows the cover of the NIRS device used in the experiments.

PEMF stimulation: To stimulate the entire right thigh, 2 circular 20 cm PEMF loop-antenna devices (Torino II **©**, Rio Grande Neurosciences, San Francisco, CA, USA) were placed on the right thigh at the level of the belly of the vastus medialis and biceps femoris caput longum (Figure 1). The PEMF waveform consisted of a pulse-burst modulated 27.12 MHz sinusoidal carrier, with 2 ms bursts width-repeated at 2 Hz and with a peak magnetic field at the center of the loop, 5 ± 1 µT.

### 2.3. Experimental Sessions

The participants visited the laboratory five times, with at least a three-day interval between each visit. The participants were instructed to refrain from moderate or intense physical activity for two days prior to each session. During each session, various recordings were performed.

On the first day (DAY 1), we assessed maximum voluntary contraction (MVC) and conducted an incremental exercise test to exhaustion in order to determine individual maximal oxygen consumption (VO_2_ max) (see Table 1). Each participant cycled on a cycle ergometer, beginning with a 5 min warm-up at 45 watts, followed by a workload starting at 60 watts with an instantaneous increase of 15 watts every minute. The cadence was maintained at 70 RPM until volitional exhaustion. Heart rate was continuously monitored using a Polar heart rate monitor (Polar Electro, Kempele, Finland).

MVC recording: Each participant performed, for five seconds, an isometric contraction against a maximum load using gym machines (Exere Air Machine, Italy). We used a leg extension machine for RVM and a leg curl for RBF. For the MCV of RBF, the subject started from a prone position, with the weight pads on the heels. For RVM, the subject started from a sitting position, with the weight pads on the ankles and a knee angle of 110°. We provided verbal encouragement in order to encourage each participant to push their limits. They repeated the same procedure 3 times, separated by 2 min of rest, and we used the MVC peak value of each investigated muscle to normalize electromyographic data (procedure already used in previous studies [22], that is, the maximum value recorded by the electromyograph during maximum voluntary contraction).

VO_2_ max recording: The subjects performed an incremental test on a cycle-ergometer (H-300-R Lode) to find the maximum oxygen consumption (VO_2_ max) of each subject, necessary to individualize the correct workload to be used for the exercise sessions. Expired gases were analyzed using a Quark b2 breath-by-breath metabolic system (Cosmed, Albano Laziale, Italy) [23]. The system was calibrated immediately before each test in accordance with the manufacturer’s guidelines: volume calibration was performed at different flow rates with a 3 L calibration syringe, and the calibration of gas analyzers was performed with a tank of reference gas mixture (16.00% O_2_, 5.00% CO_2_) and ambient air (20.93% O_2_ and 0.04% CO_2_).

From days two to four (DAY 2–DAY 4), the participants attended exercise sessions in the laboratory under two experimental conditions, PEMF ON and PEMF OFF, where the PEMF device was, respectively, activated or deactivated. Each stimulation condition lasted 15 min and was repeated twice on separate days, resulting in a total of four recordings per participant. The order of experimental sessions was randomized in advance, and all participants followed different sequences. The two PEMF loops were positioned on the right leg for both experimental conditions.

**Figure 2:**
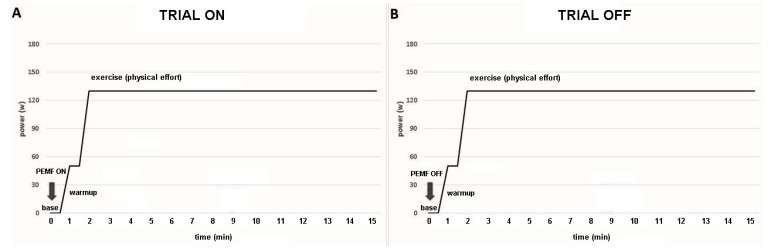
Temporal sequence of the trial. (**A**) ON trial: PEMF was activated at the beginning of the baseline (base) for 17 min (1 min baseline, 1 min warm-up, and 15 min of exercise). (**B**) OFF trial: PEMF was inactivated (sham stimulation).

Data were collected across three distinct phases: (1) baseline, (2) warm-up, and (3) constant-load exercise (Figure 2). Baseline measurements were obtained with participants seated and the right leg extended for one minute. The warm-up phase consisted of one minute of unloaded cycling. Subsequently, the workload was rapidly increased within approximately three seconds, marking the onset of the constant-load exercise phase. The participants were instructed to maintain a cycling cadence of 70 revolutions per minute (RPM) throughout the 30 min exercise period, after which the trial concluded.

EMG data were collected at baseline, warm-up (unloaded cycling), and in constant-load exercise in both experimental conditions (PEMF ON and PEMF OFF) (Figure 2). The response of muscular activity over the entire trial was recorded by surface EMG and assessed by measuring the root mean square (RMS) normalized to the peak of the MVC. In addition, we collected data regarding VO_2_ during baseline, warm-up, and exercise for each experimental condition (PEMF ON vs. OFF).

**Figure 3:**
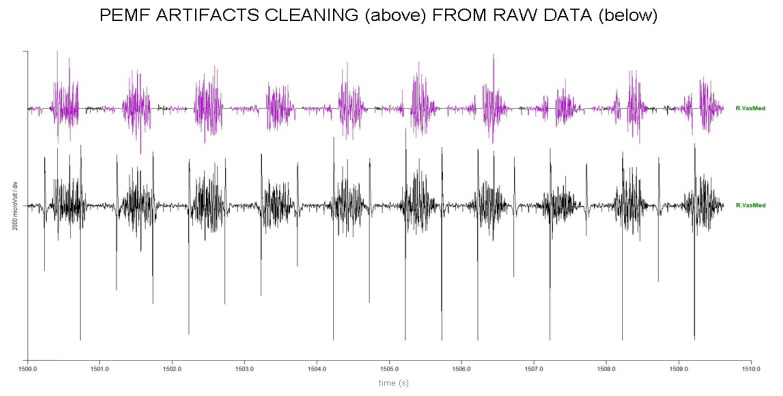
Artifacts removing process in 10 s of EMG raw trace of RVM. Upper part, purple: clean raw PEMF trace. Bottom part: original trace with PEMF artifacts before removal.

**Figure 4:**
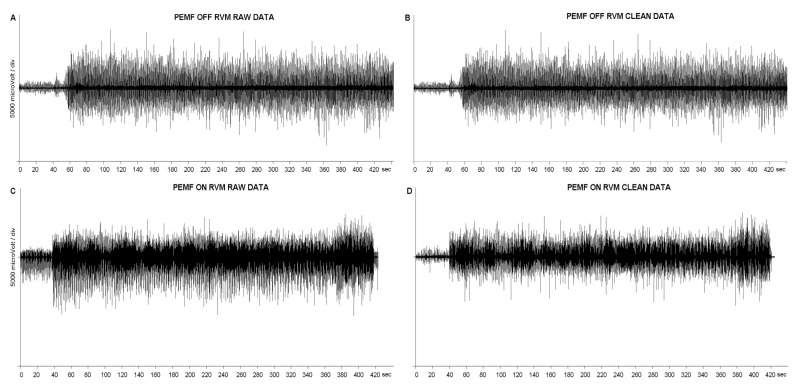
Complete EMG traces of RVM, in PEMF ON and OFF conditions, before and after the artifacts removing process.

Removing large artefacts for sEMG using low-pass filters can be risky when the artefacts’ frequency content is superimposed on sEMG data, as it may lead to the presence of spurious bursts that look like muscle activity. To avoid this risk, we used an algorithm that forces at zero a small portion of data (100 ms) surrounding the artefact peak (Figure 3 and Figure 4). This consists of four steps:

Raw data were processed using a well-known peak-enhancing procedure called Smoothed Non-linear Energy Operator (SNEO). This was introduced in the 90s to identify the QRS complex in ECG data [24] and further applied to sEMG data [25]. The output of this procedure is a non-negative amplitude profile, with the peak enhanced.

1. This amplitude profile was numerically differentiated to further separate peaks from the smoothed EMG profile, the latter being significantly reduced in amplitude by differentiation.
2. Peaks were then identified using an amplitude threshold, set at 1/8 of the global maximum, and requiring a minimum distance between consecutive peaks, set at 400 ms, i.e., the 80% of the stimulation period of 500 ms. This selection could introduce false positive detections, managed in the following step, but prevent false negative detections.
3. The time distance between peaks was used to refine the peak identification iteratively. The average stimulation frequency was prompted for a first visual verification (2 Hz expected). Then, peak locations at a distance lower than 475 ms (5% tolerance) were iteratively removed. Finally, missing stimuli locations were added in the case of a between-stimulus time distance between 525 and 975 ms (i.e., 2 × 500 − 25 ms). Once these precise artifact positions were obtained, the parts of raw data 20 ms before and 80 ms after peaks were forced to zero.

After artefact removal, muscle onset periods were identified [26], and the RMS amplitude during onsets was computed and exported in a CSV file. The whole procedure for artefact removal and RMS computation over the onset period is available as a tool in the commercial software EMG Easy Report, version 6.03.8 (Merlo Bioengineering, Parma, Italy) [27,28].

Finally, RMS values were normalized to the peak of the MVC contraction for each muscle of each subject to allow for further analysis. We averaged the values of baseline, warm-up, and constant-load exercise for each experimental condition (PEMF ON and PEMF OFF) in each investigated muscle (RBF and RVM). The data of one subject exhibited significant artifact contamination; so, the analysis was performed on eight subjects. This was still consistent with the G∗power results (see Methods Section). A repeated measures ANOVA, with baseline, warm-up, and condition (PEMF ON and PEMF OFF) as the within-subjects’ factors, was applied to both RVM and RBF muscles. Means were considered significantly different at *p* < 0.05.

## 3. Results

Data for muscle activity are presented as mean ± SD. The analysis showed a main effect of PEMF for both muscles (RVM: F_1,16_ = 10.838; *p* < 0.001; np^2^ = 0.798; RBF: F_1,16_ = 7.636; *p* = 0.003; np^2^ = 0.735). The baseline period did not show any significant differences between conditions (ON vs. OFF) for both muscles.

**Figure 5:**
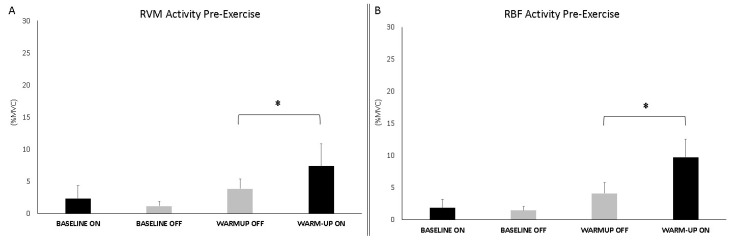
Bar graphs represent the root mean square (RMS) of the normalized EMG values of the RVM (**A**) and RBF (**B**) at the baseline and during warm-up across experimental conditions (PEMF ON vs. OFF). Data are shown as mean ± SD. The asterisk indicates significant differences at *p* < 0.05.

The analysis during warm-up showed significant differences between conditions (ON vs. OFF) for both muscles (RVM: F_1,16_ = 7.034; *p* = 0.019; np^2^ = 0.334; RBF: F_1,16_ = 23.912; *p* < 0.001; np^2^ = 0.631). Baseline and warm-up data are shown in Figure 5.

**Figure 6:**
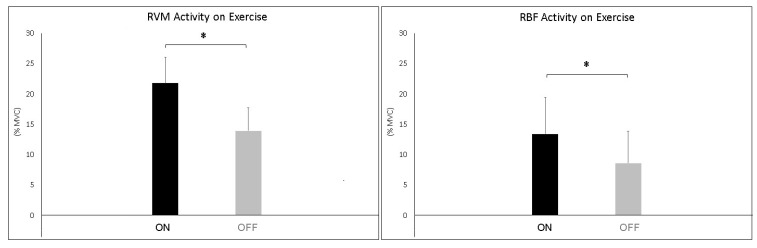
Bar graphs represent the root mean square (RMS) of the normalized EMG values of the RVM and RBF during constant-load exercise across experimental conditions (PEMF ON vs. PEMF OFF). Data are shown as mean ± SD. The asterisk indicates significant differences at *p* < 0.05.

**Figure 7:**
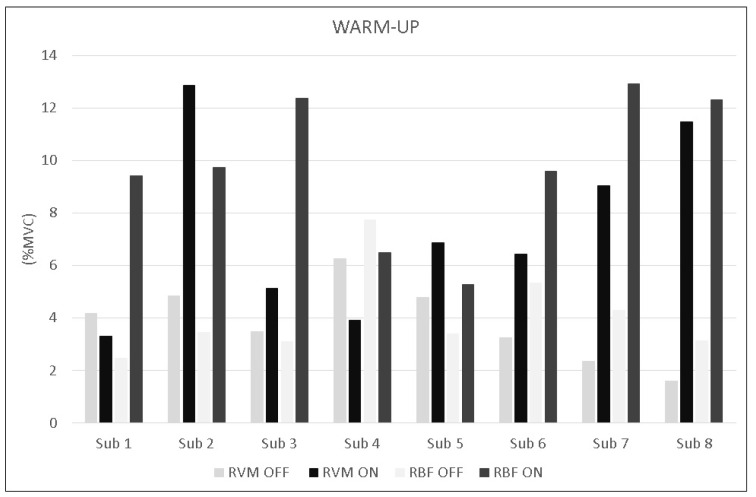
Bar graphs represent the root mean square (RMS) of the normalized EMG values of the RVM and RBF for each subject during warm-up across experimental conditions (PEMF ON vs. PEMF OFF).

**Figure 8:**
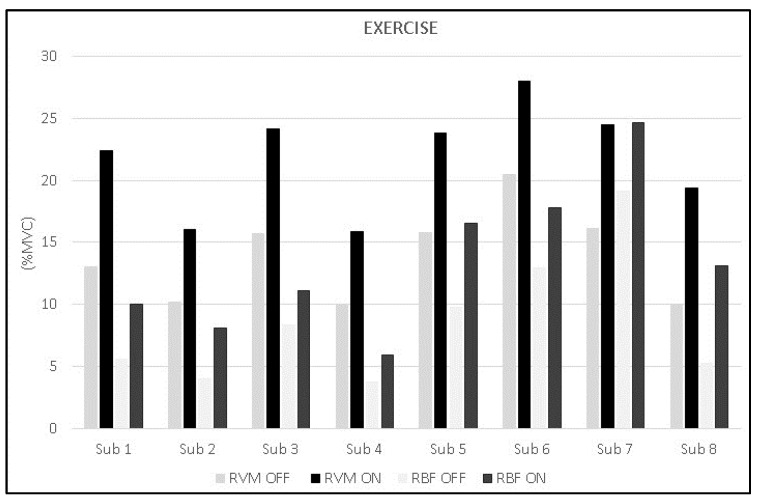
Bar graphs represent the root mean square (RMS) of the normalized EMG values of the RVM and RBF for each subject during exercise across experimental conditions (PEMF ON vs. PEMF OFF).

The analysis during constant-load exercise showed significant differences between conditions (ON vs. OFF) only for the RVM (F_1,16_ = 14.999; *p* = 0.002; np^2^ = 0.517). Figure 6 shows the average data of the exercise phase for both muscles. Data from single subjects in all conditions are shown in Figure 7 and Figure 8 for warm-up and exercise, respectively.

## 4. Discussion

The present study evaluated the acute effects of pulsed electromagnetic field (PEMF) stimulation on neuromuscular activity during moderate-intensity cycling exercise in sedentary young males. The results demonstrate that PEMF stimulation significantly increased the surface electromyographic (sEMG) amplitude in the vastus medialis (RVM) and biceps femoris (RBF) muscles, particularly during the warm-up phase and constant-load exercise. These findings provide further insight into the neuromodulatory potential of PEMF therapy and its application in exercise settings.

### 4.1. Physiological Interpretation

The primary aim of the study was to assess whether acute PEMF stimulation could enhance neuromuscular activity during moderate-intensity exercise in sedentary individuals. The significant increase in normalized EMG amplitude under PEMF stimulation, especially during the warm-up and in the RVM during constant-load cycling, suggests a facilitation of motor unit recruitment. This is particularly relevant in sedentary individuals, where muscle responsiveness and efficiency are typically diminished.

The rise in the magnitude of the muscular response induced by stimulation could be explained by the influence of PEMF on the contraction mechanism of muscular fibers [16,17]. During exercise, a crucial role is played by Ca^2+^ channels and ions flux that affects the muscular contraction. PEMF stimulation raises Ca^2+^ intracellular concentration [18,19], amplifying signal Ca^2+^ mediators, and Ca-dependent pathways [29]. Changes in membrane permeability [16] and ion channel conduction [17] might be possible mechanisms by which the pulsed electromagnetic field affects biological systems. PEMF stimulation could affect calcium channels and raise Ca^2+^ intracellular concentration [18,19], leading to improved contraction mechanisms. Furthermore, PEMF stimulation could amplify signal Ca^2+^ mediators and Ca^2+^-dependent pathways, probably through changes in the phospholipids of the plasma membrane [30,31,32].

The analysis showed a significantly higher activity for RVM with respect to RBF. This result seems coherent with the effective and well-known role of vastus medialis during cycling. On the other hand, the role of bicep femoris is still unclear as its effective action is more affected by fatigue, pedaling rate, coordination/activation timing (angle), training status, shoe–pedal interface, and body position [33]. During cycling, the biceps femoris seems to be more important for energy transfer between joints rather than to supply the main force [33]. The earliest activation and largest activity of bicep femoris seem to be related to increased fatigue in vastus lateralis and vastus medialis as a consequence of modified activation patterns and coordination [33]. In this study, the moderate workload caused a large activity of the vastus medialis, which is the main muscle of cycling, and a low activation of the biceps femoris. These results support the use of PEMF as a neuromodulatory tool that could enhance the quality of muscle activation during the initial stages of physical activity, potentially improving training efficiency and reducing injury risk associated with poor muscle readiness. The improved activation observed in both primary (RVM) and synergist (RBF) muscles suggests that PEMF may broadly enhance motor drive during submaximal exercise, supporting its utility as a warm-up adjunct.

The increased EMG amplitude under PEMF stimulation likely reflects several converging physiological mechanisms. First, the PEMF-induced enhancement of calcium influx via voltage-gated calcium channels may facilitate the binding of Ca^2+^ to calmodulin, subsequently activating endothelial nitric oxide synthase (eNOS) and stimulating NO production. This cascade promotes vasodilation, increases tissue oxygenation, and supports muscle fiber recruitment. Second, changes in cellular membrane permeability and modulation of ion flux, particularly sodium and potassium, may alter the excitability threshold of motor units, resulting in more efficient recruitment. These effects are consistent with previous studies showing increased median frequency and shortened electromechanical delay under PEMF, suggesting improved neuromuscular transmission and contractile efficiency [8].

Finally, while systemic metabolic markers (e.g., VO_2_, lactate) were not analyzed in the present study, prior research indicates that PEMF may enhance glycolytic activity in type II fibers and influence local lactate dynamics [14,15]. The EMG patterns observed in this paper may thus also reflect improved metabolic support during sustained activity.

### 4.2. Comparison with the Existing Literature on PEMF and Exercise

The observed enhancements in muscle activation under PEMF stimulation align with earlier studies reporting improved local oxygen kinetics, vasodilation, and neuromuscular performance under PEMF conditions. Trofè et al. [14,15] previously found that PEMF improved muscle oxygenation and deoxyhemoglobin kinetics in trained cyclists during constant-load exercise, supporting our findings of increased EMG amplitude during active muscular engagement. Similarly, Jeon et al. [8] reported improved muscle recruitment and reduced electromechanical delay during isometric contraction following PEMF treatment applied to the biceps brachii, albeit with no changes in peak force output.

Our results also align with the review by Ghanbari Ghoshchi et al. [34], which concluded that PEMF stimulation can acutely enhance muscle activation and tissue perfusion by modulating calcium signaling and nitric oxide (NO) production. The increase in RMS amplitude we observed may reflect a similar physiological mechanism.

However, the findings contrast with Granja-Domínguez et al. [35], who found no significant improvement in fatigue, gait performance, or quality of life in individuals with relapsing–remitting multiple sclerosis following a four-week PEMF intervention. Differences in population (neurologically impaired vs. healthy), stimulation parameters, and outcome measures likely account for this discrepancy. Additionally, Ong et al. [36] proposed a mitochondrial mechanism for PEMF-induced muscle strength gains in postoperative anterior cruciate ligament patients. Although their study is ongoing, our findings provide preliminary empirical support for these proposed mechanisms in a healthy population.

Considering the use of PEMF in human studies, an important consideration has to be made in regards to the stimulation parameters, given that the accurate interaction between PEMF and human cells and tissue is still unclear. Further studies need to be performed with different PEMF timings and frequencies, because the different responses may depend on the dosage of stimulation of a specific electromagnetic signal in addition to the specific biological tissue [37].

### 4.3. Limitations

While the study design employed rigorous controls, including randomized, single-blind trials, certain limitations merit consideration.

All participants were young sedentary males. The aim of this study was strongly focused on investigating the effects of PEMF on muscle activity during exercise, excluding additional variables. Thus, we recruited only male participants, given that the menstrual cycle could affect physical and sports activity [38,39,40]. In further studies, it will be interesting to investigate the effects of PEMF stimulation on young sedentary females to evaluate the potential gender differences in PEMF stimulation on muscle activity, especially during the different phases of the menstrual cycle.

The acute experimental protocol assessed muscle activity during single sessions; thus, the long-term effects of repeated PEMF exposure on muscle function and exercise performance remain unknown. Longitudinal studies incorporating training adaptations and functional outcomes like strength, endurance, or recovery metrics should be further investigated.

While EMG amplitude is a useful proxy for muscle activation, it cannot fully capture underlying physiological changes such as muscle fiber recruitment patterns, metabolic alterations, or vascular responses. Combining EMG with complementary techniques (e.g., muscle biopsy or biochemical assays) would strengthen mechanistic interpretations.

In this experiment, we used only one constant workload throughout the duration of the exercise. Further studies could investigate the effects of PEMF stimulation at different workloads, especially between 50% of VO_2max_ and the ventilatory threshold (VT), to evaluate the relationship between PEMF and exercise intensity.

Another limitation concerns the anthropometric characteristics of the subjects. In this study, we enrolled only normal- or light overweight people. Further studies could investigate the effects of PEMF stimulation on subjects with different BMI values (i.e., sedentary subjects with obesity) to determine if the PEMF stimulation can increase the metabolic activity of the muscles in all populations.

Lastly, although the sample size in this study is relatively small, the findings offer valuable preliminary insights into the phenomenon under investigation. The focused sample allows for a detailed and controlled exploration, which can inform and guide subsequent larger-scale studies. The results contribute meaningfully to the existing body of knowledge and highlight important trends worthy of further investigation. Future research with expanded samples will be essential to validate and build upon these promising initial findings.

### 4.4. Theoretical Implications and Future Directions

Our findings support the theoretical framework that PEMF can modulate neuromuscular excitability and muscle metabolic processes, enhancing muscle recruitment during exercise. This aligns with the growing recognition of PEMF as a non-invasive modality capable of influencing cellular and tissue function beyond pain management and tissue healing.

Given the moderate to large effect sizes observed, PEMF could potentially be integrated as a novel ergogenic aid or therapeutic intervention in populations with reduced muscle function or during rehabilitation. Nevertheless, more comprehensive studies are needed to elucidate the dose–response relationships, optimal stimulation parameters, and the interaction between PEMF and various exercise modalities.

This study contributes novel evidence encouraging the further exploration of PEMF as a promising tool to improve muscle function, with implications spanning athletic performance, clinical rehabilitation, chronic pathologies, and healthy aging.

## 5. Conclusions

This study demonstrates that pulsed electromagnetic field (PEMF) stimulation acutely enhances muscle activation in the vastus medialis and, to a lesser extent, the biceps femoris during moderate-intensity cycling exercise in sedentary young men. The increased electromyographic activity observed suggests that PEMF may facilitate neuromuscular excitability and muscle recruitment, potentially through mechanisms involving calcium signaling and improved muscle perfusion. While these findings support the promising role of PEMF as a non-invasive modality to augment muscle function during exercise, further research with larger, more diverse populations and longer-term interventions is needed to fully understand its physiological effects and practical applications. Overall, PEMF represents a valuable avenue for future exploration in both sports performance enhancement and clinical rehabilitation contexts.

## Data Availability

The data that support the findings of this study are not openly available due to reasons of sensitivity and are available from the corresponding author upon reasonable request. Data are located in controlled access data storage at the University of Bologna (Italy).
